# Frequency and Amount of Caffeine Consumption and Its Effects on Sleep Quality Among the General Population in Makkah City, Saudi Arabia, in 2024

**DOI:** 10.7759/cureus.65730

**Published:** 2024-07-30

**Authors:** Ruolan M Khan, Jehad Alqurashi

**Affiliations:** 1 Preventive Medicine, Saudi Board of Preventive Medicine, Makkah, SAU; 2 Public Health, Ministry of Health, Saudi Board of Preventive Medicine, Makkah, SAU

**Keywords:** sleep health, sleep deprivation, psychoactive substance, pittsburgh sleep quality index (psqi), caffeine intake

## Abstract

Background: Caffeine is a commonly consumed psychoactive substance with potential effects on sleep quality. However, few studies have examined this relationship in the general population of Makkah, Saudi Arabia.

Objective: To investigate the impact of caffeine consumption on sleep quality and identify demographic factors associated with caffeine intake and sleep quality in Makkah's general population.

Methods: This cross-sectional study used a convenient sampling approach to recruit 390 individuals residing in Makkah. Data were collected via a Google Forms questionnaire distributed through various social media platforms. Caffeine intake and sleep quality were assessed using a validated questionnaire adapted for Saudi Arabia and the Pittsburgh Sleep Quality Index (PSQI), respectively.

Results: The median total daily caffeine intake was 324.20 mg, with 43.1% of participants consuming more than the suggested cutoff of 400 mg daily. There were significant positive correlations between PSQI and total caffeine consumption in the morning, evening, and overall daily consumption. The results also indicated significant relationships between demographic factors, such as marital status, educational level, income, and BMI groups, and caffeine consumption.

Conclusion: Caffeine intake was found to be high among the general population of Makkah, with a significant portion consuming more than the suggested daily limit. There was a significant positive correlation between caffeine consumption and poor sleep quality. Additionally, demographic factors such as marital status, educational level, income, and BMI groups were found to be associated with caffeine consumption.

## Introduction

Caffeine is the most widely used psychoactive substance worldwide, with an 80% consumption rate [1]. It is commonly found in coffee, as well as other foods and beverages such as coca and energy drinks [2]. Caffeine is used to reduce sleepiness and improve performance. Pharmacologically, caffeine acts as an adenosine-receptor antagonist [2]. Therefore, its performance benefits are largely due to its ability to block adenosine receptors (AR) [2]. Caffeine primarily affects AR1 and AR2A, which are associated with brain processes related to sleep, arousal, and cognition [3]. However, despite its performance advantages, caffeine consumption is often associated with poor sleep quality [4].

Although there is no universal agreement on how to define sleep quality, it can generally be described as sleep contentment. This definition takes into account both objective and subjective aspects of sleep [5]. While objective measurement of sleep quality can be challenging, an easy and fast tool has been introduced to help measure sleep quality. The Pittsburgh Sleep Quality Index (PSQI) has been used to measure aspects related to sleep quality [6].

The consumption of caffeine has been associated with demographic variables such as sex, as well as behaviors such as drinking alcohol and smoking [7]. Studies have shown a higher prevalence of caffeine consumption among women [8]. Previous studies have also found that smokers consume more caffeine than non-smokers in the general population [9]. It is worth noting that smoking increases the primary pathway for caffeine metabolism, suggesting that smokers may need to consume more caffeine to achieve the same effects as non-smokers [10]. The motivation to experience both the benefits of daytime alertness and a good subjective quality of sleep despite prior stimulant use likely explains the typical pattern of caffeine consumption in the morning and afternoon [11,12].

Caffeine's impact on the homeostatic aspect of sleep-wake regulation is believed to be responsible for both its alerting effects and its disruptive effects on sleep [13]. Higher PSQI scores indicate poorer sleep quality, which has been linked to various chronic diseases such as type 2 diabetes, heart disease, obesity, and depression, as well as accidents and mistakes in the workplace that result in damage and disability [14]. It is widely recognized that sleep deprivation leads to significant declines in cognitive performance, including decreased attention, alertness, vigilance, and slower cognitive and psychomotor responses. These connections demonstrate that poor sleep quality has a significant impact on the global burden of disease and provides an opportunity for public health intervention [15].

Numerous studies have assessed sleep quality both nationally and internationally [7,16-19]. In fact, higher rates of depression and anxiety symptoms were noticed among patients with low sleep quality [18]. Studies done in Saudi Arabia have concentrated on particular populations with common medical issues or tiny communities, such as college students [19]. The relationship between poor sleep quality and caffeine consumption in the general population has not been adequately examined in any of the earlier Saudi studies. This is mainly due to the scarcity of results and difficulty in accurately assessing caffeine consumption. Thus, the study's objective is to explore the relationship between caffeine consumption and the perceived quality of sleep among the general population in Makkah, Saudi Arabia.

## Materials and methods

Study design 

This study employed a cross-sectional, correlational design to investigate the relationship between caffeine consumption and sleep quality among the general population in Makkah City, Saudi Arabia.

Area and population 

The study was conducted in Makkah City, Saudi Arabia, targeting the general population residing in the area. Efforts were made to invite a diverse sample of the target population in terms of age, gender, and socioeconomic status to enhance the generalizability of the results. Adults of both genders, residing in Makkah City, who had access to the questionnaire were invited to participate in the study. The study included individuals aged >18 years old who were residents of Makkah City. There were no specific exclusion criteria, allowing for a broad representation of the general population.

A non-probability convenient sampling approach was utilized to recruit participants for this study. The sample size was determined based on the feasibility and resources available for data collection. The sample size calculation was conducted using an online website (Raosoft.com: http://raosoft.com/). The formula considered a 95% confidence interval, a 5% margin of error, and a prevalence of poor sleep quality of 50%. The resulting sample size was determined to be 384 patients.

Data collection tools and technique

The data collection process was facilitated by an electronic form designed on Google Forms. The participants were provided with a link to access the questionnaire and instructed to complete it at their convenience. The link was distributed through various social media platforms, such as WhatsApp, X-app (previously Twitter), and Telegram. The data collection period took place from 20 February to 31 May 2024.

We collected data on caffeine consumption using a validated questionnaire specifically designed to assess caffeine intake [20]. The questionnaire was adapted to include caffeine beverages and forms of intake relevant to Saudi Arabia. The key variables of interest in this study were caffeine intake and sleep quality. Caffeine intake, for our purposes, refers not only to the consumption of coffee but is defined to include other sources of caffeine, such as tea, energy drinks, and certain types of soft drinks. To enhance the accuracy of reported coffee intake, the questionnaire included photographs demonstrating various cup sizes. Sleep quality was measured using the PSQI. The PSQI is a validated instrument designed to assess a range of sleep parameters and disturbances over a one-month interval, providing a nuanced view of the participants' sleep quality [21]. This tool enabled us to capture both the objective and subjective aspects of sleep quality, contributing to a more well-rounded understanding of sleep health among the population. The independent variable was caffeine intake, which referred to the amount and sources of caffeine consumed by the participants, including beverages such as coffee, tea, energy drinks, and caffeine-containing soft drinks such as Cola products. The dependent variable was sleep quality, as assessed using the PSQI. This measure included subjective aspects of sleep quality, such as subjective sleep quality, sleep latency, sleep duration, sleep efficiency, sleep disturbances, use of sleep medication, and daytime dysfunction.

Scoring and cutoff

The scoring and cutoff criteria for the PSQI questionnaire adhered to the original instrument developers' guidelines. A score of five or more indicated poor sleep quality [21]. In contrast, the cutoff criteria for caffeine intake were established based on recommendations from relevant health authorities and prior studies. Therefore, a daily caffeine intake of 400 mg was used to categorize participants into different intake groups (e.g., low, high) [22]. This cutoff helped assess the potential impact of different levels of caffeine intake on sleep quality among the general population in Makkah City, Saudi Arabia. Daily caffeine was calculated based on the participants' entry of drunken amount. Daily caffeine intake, calculated separately for morning and evening intake and summed for a daily total (in milligrams), was used for primary analyses. The calculation and units were primarily made according to the caffeine chart published by the Center for Science in the Public Interest [23].

Statistical analysis 

Statistical analysis was conducted using Statistical Product and Service Solutions (SPSS, version 29.0; IBM Corp., Armonk, NY). The normality of continuous variables was tested with the Shapiro-Wilk test, and due to skewness, these variables were summarized using median and interquartile range (IQR). Categorical variables were summarized using frequency tables and proportions. A cutoff of 400 mg was applied to create a binary variable for group comparisons. Chi-square tests were used to examine the associations between demographic and work-related variables and the daily caffeine intake cutoff. Spearman’s correlation was employed to determine the direction and strength of the association between PSQI and daily caffeine intake. A p-value less than 0.05 was considered statistically significant for all inferential tests.

Ethical considerations 

Ethical approval was obtained from the institutional review board. Informed consent was obtained from all study participants prior to data collection, ensuring confidentiality and anonymity. Participants were provided with detailed information about the study's nature and objectives. Participation was voluntary, and participants could withdraw at any time without consequences. Data were securely stored and accessed only by authorized researchers, and all personal identifiers were removed to ensure anonymity and protect participants' privacy.

## Results

In this study, a total of 390 responses were collected. The age of the participants ranged from 18 to 88 years, with a median of 33 (IQR: 26-43). The sample was almost equally distributed between males (48.2%) and females (51.8%). The majority of the participants were Saudi nationals (89.7%). In terms of marital status, 52.8% were married, 34.6% were single, 8.7% were divorced, and 3.8% were widows. The educational level of most participants was a bachelor's degree (51.3%). Income levels varied, with 29.5% earning more than 15,000 Saudi Riyals (SAR), 21.8% earning 10,001-15,000 SAR, 27.2% earning 5,001-10,000 SAR, and 21.5% earning less than 5,000 SAR. The majority of the participants had a normal BMI (44.1%), and 65.1% did not smoke. Regarding comorbidity, the majority reported no conditions (64.9%). The results are shown in (Table 1).

**Table 1:** Demographic characteristics and caffeine consumption of the participants *P-value calculated using the chi-square test or Exact test

Variable	Groups		Total (%)	Caffeine Consumption		P-value
				≤ 400 mg	>400 mg	
Gender		Male	188 (48.2%)	99 (52.7%)	89 (47.3%)	0.103
		Female	202 (51.8%)	123 (60.9%)	79 (39.1%)	
Nationality		Saudi	350 (89.7%)	198 (56.6%)	152 (43.4%)	0.738
		Non-Saudi	40 (10.3%)	24 (60%)	16 (40%)	
Marital status		Single	135 (34.6%)	65 (48.1%)	70 (51.9%)	0.030*
		Married	206 (52.8%)	122 (59.2%)	84 (40.8%)	
		Divorced	34 (8.7%)	24 (70.6%)	10 (29.4%)	
		Widow	15 (3.8%)	11 (73.3%)	4 (26.7%)	
Educational level		No official education	13 (3.3%)	2 (15.4%)	11 (84.6%)	<0.001*
		Less than high school	26 (6.7%)	9 (34.6%)	17 (65.4%)	
		High school	43 (11%)	18 (41.9%)	25 (58.1%)	
		Diploma	24 (6.2%)	9 (37.5%)	15 (62.5%)	
		Bachelor	200 (51.3%)	137 (68.5%)	63 (31.5%)	
		Higher education	84 (21.5%)	47 (56%)	37 (44%)	
Income (Saudi Riyals)		≤5,000	84 (21.5%)	33 (39.3%)	51 (60.7%)	0.003*
		5,001-10,000	106 (27.2%)	64 (60.4%)	42 (39.6%)	
		10,001–15,000	85 (21.8%)	52 (61.2%)	33 (38.8%)	
		>15,000	115 (29.5%)	73 (63.5%)	42 (36.5%)	
BMI groups		Underweight	26 (6.7%)	9 (34.6%)	17 (65.4%)	0.037*
		Normal	172 (44.1%)	93 (54.1%)	79 (45.9%)	
		Overweight	117 (30%)	73 (62.4%)	44 (37.6%)	
		Obesity class I	52 (13.3%)	34 (65.4%)	18 (34.6%)	
		Obesity class II	17 (4.4%)	9 (52.9%)	8 (47.1%)	
		Obesity class III	6 (1.5%)	4 (66.7%)	2 (33.3%)	
Smoking		Yes	136 (34.9%)	73 (53.7%)	63 (46.3%)	0.391
		No	254 (65.1%)	149 (58.7%)	105 (41.3%)	

The total caffeine consumption was analyzed at different times of the day and overall. The total caffeine consumption during the daytime (08:00-16:00) ranged from 0.00 mg to 7,777 mg, with a median of 250 mg (0-565 mg). In the evening time (after 16:00), total caffeine consumption showed a notable decrease with a range from a minimum of 0.00 mg to a maximum of 7,690 mg, with a median of 16.40 mg (IQR: 0-381 mg). However, the daily total caffeine consumption ranged from a minimum of 0.00 mg to a maximum of 10,050 mg, with a median of 324.20 mg (IQR: 114.20-970.20 mg). The daily intake was further divided using a cutoff of 400 mg. This revealed that 43.1% of participants consumed more than 400 mg of caffeine daily, indicating a high prevalence among our participants.

Gender did not significantly influence caffeine consumption (>400 mg) among the participants, with 47.3% of males and 39.1% of females reporting high caffeine consumption (p=0.103). Marital status was significantly associated with caffeine consumption. Among those consuming more than 400 mg of caffeine daily, 51.9% among single, 40.8% among married, 29.4% among divorced, and 26.7% among widowed (p=0.030). Educational level was significantly associated with caffeine consumption, with the highest consumption reported among those with no official education (84.6%), followed by less than high school (65.4%), high school (58.1%), diploma (62.5%), bachelor's degree (31.5%), and higher education (44%) (p<0.001). Income level was significantly associated with caffeine consumption, with the highest consumption reported among those earning ≤5,000 SAR (60.7%) and the lowest among those earning >15,000 SAR (36.5%) (p=0.003). BMI groups were significantly associated with caffeine consumption, with the highest consumption reported by underweight individuals (65.4%), compared to a lower prevalence of high caffeine consumption among obesity classes (34.6%-47.1%; p=0.037). Smoking status did not significantly influence caffeine consumption, with 46.3% of smokers and 41.3% of non-smokers consuming more than 400 mg of caffeine daily (p=0.391). The detailed results are shown in (Table 1).

In terms of employment status, 62.8% of the participants were employed, and 37.2% were not. When asked about their work schedule, 56.2% reported having a constant-hours job, while 43.8% did not. The work times varied among the participants, with 45.9% working in the morning, 6.9% in the evening, 1.5% at night, and 45.6% not working at a specific time. In terms of work nature, 45.6% had a regular job, 6.2% worked shifts, 9.5% were on-calls, 3.8% had other work nature, and 34.9% did not specify their work nature. As for breakfast habits, 31.5% of the participants reported regularly eating breakfast. Timing of breakfast varied, with 14.4% eating within 30 minutes of waking up, 33.2% within one hour, 24.6% within two hours, and 27.8% eating breakfast after two hours. Our analyses showed a significant correlation between employment status and daily caffeine intake (p=0.020). Among employed participants, 47.8% consumed more than 400 mg, compared to 35.2% among those who were unemployed. Additionally, work timings significantly influenced caffeine consumption (p<0.001). Among those working in the morning, 40.8% consumed more than 400 mg. Meanwhile, all of the participants who worked at night (n=6) consumed more than 400 mg of caffeine. The nature of work also played a role in caffeine consumption (p=0.002). Among those with regular jobs (n=178), 41% consumed more than 400 mg daily, compared to 75% among shift workers. Regarding breakfast habits, although the p-value was not significant, among those who regularly ate breakfast, 49.6% consumed more than 400 mg of caffeine, compared to 40.1% among those who reported skipping breakfast meals. The results are further detailed in Table 2.

**Table 2:** Distribution of caffeine consumption and sleep quality across demographic variables *P-value calculated using the chi-square test or Exact test

Variable		Groups	Total (%)	Daily Caffeine Consumption		P-value
				≤ 400 mg	>400 mg	
Employment status	Employed		245 (62.8%)	128 (52.2%)	117 (47.8%)	0.020*
	Unemployed		145 (37.2%)	94 (64.8%)	51 (35.2%)	
Do you have a constant hours job?	Yes		219 (56.2%)	125 (57.1%)	94 (42.9%)	0.944
	No		171 (43.8%)	97 (56.7%)	74 (43.3%)	
Work time	Morning		179 (45.9%)	106 (59.2%)	73 (40.8%)	<0.001*
	Evening		27 (6.9%)	8 (29.6%)	19 (70.4%)	
	Night		6 (1.5%)	0 (0%)	6 (100%)	
	None		178 (45.6%)	108 (60.7%)	70 (39.3%)	
Work nature	Regular		178 (45.6%)	105 (59%)	73 (41%)	0.002*
	Shifts		24 (6.2%)	6 (25%)	18 (75%)	
	On-calls		37 (9.5%)	16 (43.2%)	21 (56.8%)	
	Other		15 (3.8%)	7 (46.7%)	8 (53.3%)	
	None		136 (34.9%)	88 (64.7%)	48 (35.3%)	
Regularly eating breakfast	Yes		123 (31.5%)	62 (50.4%)	61 (49.6%)	0.078
	No		267 (68.5%)	160 (59.9%)	107 (40.1%)	
Breakfast time	Within 30 mins		27 (14.4%)	6 (22.2%)	21 (77.8%)	0.194
	Within one hour		62 (33.2%)	27 (43.5%)	35 (56.5%)	
	Within two hours		46 (24.6%)	18 (39.1%)	28 (60.9%)	
	After two hours		52 (27.8%)	24 (46.2%)	28 (53.8%)	

**Figure 1:**
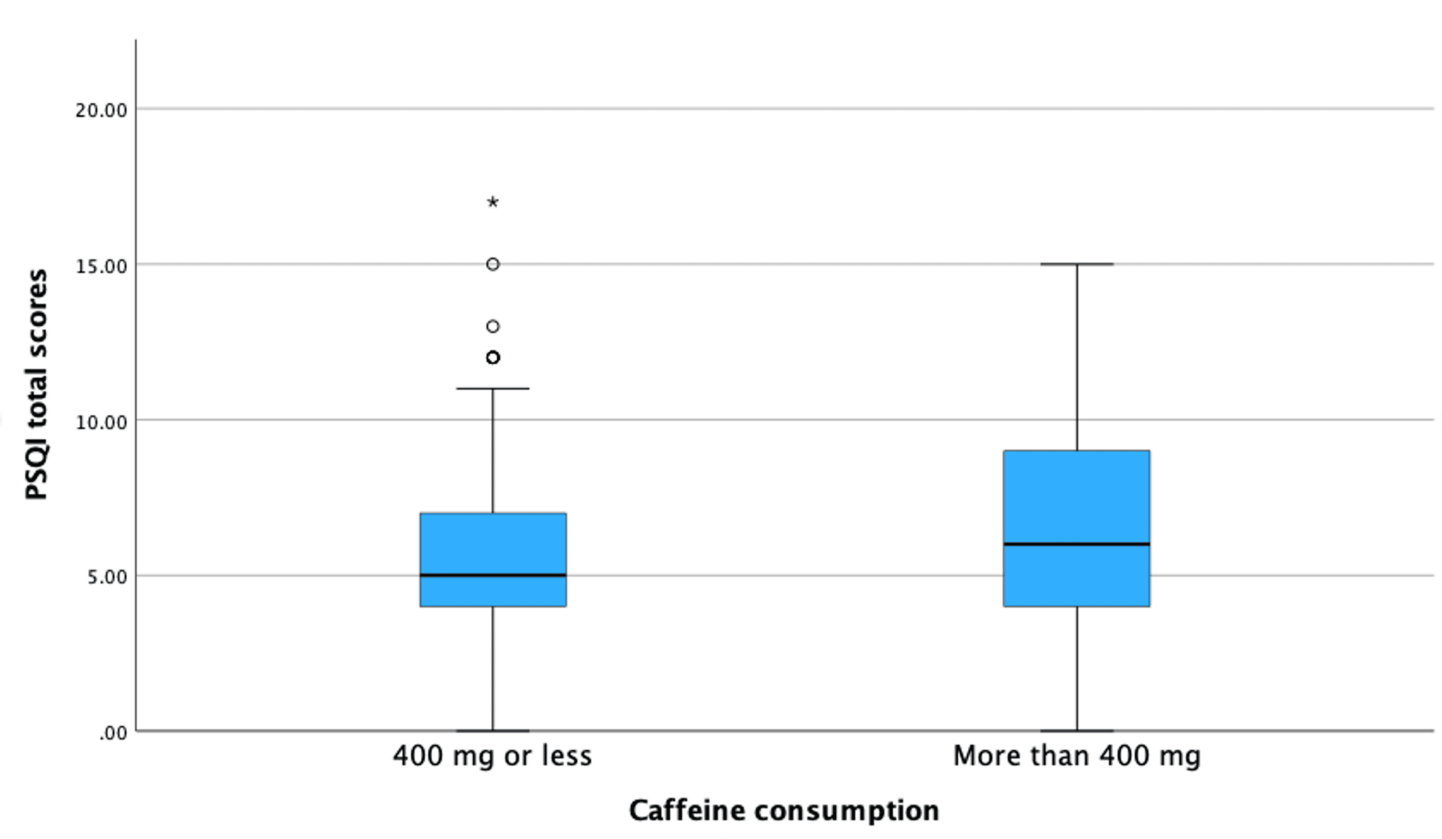
Boxplot illustrating the association between daily caffeine consumption and sleep quality represented by PSQI scores

The results of PSQI total scores ranged from zero to 17, with a median of 6 (IQR: 4-8). Upon further analyses of sleep quality, there were significant positive correlations between PSQI and total caffeine consumption in the morning (r=0.209, p<0.001), in the evening (r=0.191, p<0.001), and overall daily consumption (r=0.187, p<0.001). This suggests that, as caffeine consumption increases, sleep quality decreases. Further analysis included the analysis of total PSQI. When the total PSQI scores were analyzed with the groups of daily caffeine intake, the results showed significantly poor sleep among those with higher caffeine intake. Specifically, those who consumed more than 400 mg of caffeine daily had a median PSQI score of 6 (IQR: 4-9), compared to a median of 5 (IQR: 4-7) among those consuming less than 400 mg of caffeine daily. These findings were statistically significant (p<0.001). This finding is further demonstrated in Figure 1.

## Discussion

The primary objective of our study is to evaluate the effects of caffeine consumption on the sleep quality of the people in Makkah, Saudi Arabia. This finding makes a valuable contribution to the existing knowledge base on the subject matter, particularly within the context of Saudi Arabian society. The key finding of our study showed that the majority of the people in Makkah are consuming caffeine, with more than 43.1% taking more than the recommended daily recommended limit of 400 mg. This is higher than the global trends as surveys conducted have revealed patterns of consumption to be comparable to what is found in the United States and Western Europe [22,24]. However, it is worth noting that data included caffeine consumption from beverages, pills, chocolates, and other caffeinated products [24]. High caffeine intake was found among populations who are single (34.6%) and populations who have higher education (bachelor’s degree and above, 72.8%). Similar findings were reported by Riera-Sampol et al. [25]. Another key finding from this research was that there was a positive correlation between caffeine consumption and reduced sleep quality as measured by PSQI scores. This is consistent with previous research showing that coffee interferes negatively with sleep in different populations, including university students, in which poor quality of sleep was noted (median PSQI: 6) [16,25]. Therefore, this finding fits well into the previous body of knowledge indicating that caffeine affects subjective sleep measures such as total sleep time, sleep latency, and sleep quality [18,25]. Few studies have reported that university students have been studied and higher caffeine consumption among them most especially from soda was found to be associated with lower sleep quality scores as opposed to other caffeinated drinks [16]. It is worth noting that medical students in Saudi Arabia also have a high incidence of consuming coffee beverages and recording poor slumber results at the same time [18,26].

The observation of this study corroborates with findings of other published studies conducted among different populations globally. The effects of caffeine recorded in the studies yielded in relation to sleep duration and sleep disturbances are in line with previous research studies that involved university students and women only [16,25]. Additionally, the consistently elevated figures of poor sleep quality noted among the Saudi Arabian population signifies relatively above 70% in some of the studies [18,25,27]. This supports the future pursuit of research on these factors and the ways by which improved sleep health in the area could be encouraged. Moreover, further examination of moderating factors such as age, genetic factors, and general health status should be conducted regularly. For example, although moderate caffeine consumption is likely to help people with anxiety disorders (in general), those from this category are likely to experience sleep disturbances due to excess caffeine consumption [19]. This corroborates the finding of high levels of caffeine use and poor sleeping habits among medical students in Saudi Arabia [19]. The importance of these findings in terms of public health cannot be overemphasized, especially in light of studies conducted across the world where the prevalence of poor sleep quality was estimated to be as low as 38.2%-72.5% [18].

One of the major advantages of this study is that it has a broad and diverse sampling frame within Makkah as it lowers the chances of skew while identifying the average caffeine intake associated with average sleeping patterns in the city. Additionally, by using a standard measure such as PSQI, instead of subjective evaluations, data ambiguity was minimized, and attention was focused on sleep quality. This study employs a cross-sectional design, which inherently limits our ability to establish causal relationships between caffeine consumption and sleep quality. This design captures a snapshot in time and cannot determine which factor came first. Individuals with pre-existing poor sleep patterns might naturally consume more coffee during the day to stay awake, potentially creating a reverse causality issue. Another limitation is regarding methodology since survey questionnaires were administered and self-reported experiences were taken into consideration; these can be challenged by recall as well as social desirability biases. Finally, the convenient sampling technique also limits generalizability to the reference population.

## Conclusions

The data indicate a high prevalence of caffeine consumption, with a notable proportion of individuals consuming more than 400 mg per day. The findings demonstrate a significant association between high caffeine consumption and poor sleep quality, suggesting that reducing caffeine intake could potentially improve sleep outcomes for many individuals. Future studies should explore the underlying mechanisms linking caffeine consumption to sleep disturbances to provide a more comprehensive understanding of the observed associations. Longitudinal studies could offer insights into the long-term effects of chronic caffeine consumption on sleep health. Additionally, expanding the research to include diverse populations and age groups could help generalize the findings and develop tailored interventions to mitigate the adverse effects of caffeine on sleep quality.

## Appendices

Caffeine intake is measured from the following chart, estimated separately for day and evening [20].

**Table 3:** Measurement of morning and evening caffeine intake by beverage type and volume

	120 mL	235 mL	295 mL	355 mL	475 mL	590 mL
Saudi Coffee						
American Coffee						
Turkish Coffee						
Espresso						
Cappuccino, Frappuccino, Latte, or Cortado						
Decaf Coffee						
Other Coffee						
Tea						
Energy Drink						
Coca-Cola						
Hot Chocolate						
Other Types						
Solpadeine Tablets						
Solpadeine Tablets						
Panadol Tablets						
Fevadol Tablets						

Assessment of sleep quality using the PSQI tool [21].

**Table 4:** Pittsburgh Sleep Quality Index (PSQI) questionnaire for the assessment of sleep quality

Question		Answer			
1. When do you usually go to bed?					
2. How long (in minutes) has it taken you to fall asleep?					
3. When do you usually get up in the morning?					
4. How many hours of actual sleep do you get at night?					
5. During the past month, how often have you had trouble...					
		Not during the past = 0	Less than once a week = 1	Once or twice a week = 2	Three or more times a week = 3
a. Cannot get to sleep within 30 min -					
b. Wake up in the middle of the night or early morning					
c. Have to get up to use the bathroom					
d. Cannot breathe comfortably					
e. Cough or snore loudly					
f. Feel too cold					
g. Feel too hot					
h. Have bad dreams					
i. Have pain					
j. Other reason(s), please describe - (Scoring based on description)					
6. During the past month, how often have you taken medicine...					
7. During the past month, how often have you had trouble...					
8. During the past month, how much of a problem has it been...					
9. During the past month, how would you rate your sleep...		Very good = 0	Fairly good = 1	Fairly bad = 2	Very bad = 3
